# Editorial

**Published:** 1890-12

**Authors:** 


### Editorial.

### A MUCH NEEDED LEGISLATION.

Now that the representatives of the people are assembled at the capital in the capacity of legislators, it is in order for us to make such suggestions as will more fully acquaint them with our wishes and necessities.

Some provision ought to be made for the care-taking and reformation of the unfortunate inebriates of the State. It is a crying shame that nothing has been done in the Empire State of the South to give this greatly needed relief. The world is very uncharitable towards the victims of alcohol. Prohibitory laws have proven a failure; public sentiment is morbid on the subject. Temperance union movements have done much good in educating public opinion, but cannot reach these poor, ruined individuals. What shall be done with them? If inquiry be made, it will show that a large per cent. of the crimes committed—especially the homicides—are due to the effects of whisky, or some other alcoholic liquor. The sale of intoxicants is licensed and the revenue turned into the public treasury, but there is no provision made to reform the miserable drunkard. Why is this? It is because of the *false opinion* that is abroad that drunkenness is a *sin* but not a *disease*. Such belief obtains only among the uninformed. But we will not stop here to discuss the question. We know whereof we speak when we say that inebriety is one of the most intractable diseases known to the science of medicine. It makes a physical, mental and moral wreck of the individual, unless restored by appropriate treatment. Leaving all other questions aside, we feel that if the State of Georgia would establish and endow an asylum for these unfortunate citizens, it will prove a most economic legislation. The cost of keeping aud prosecuting criminals, and providing for the insane in the asylum, would soon diminish sufficiently to admit of the establishing of suitable hospitals for the inebriate without any increased expense to the State. Humanity cries out, the heart-broken wives cry out, and the worse than fatherless children cry out for something to be done in this behalf. Legislators have promised to make needed reforms. Here is their golden opportunity. We are confident that Governor Northen will stand by them; that the people will give them their hearty endorsement, and when they return to their homes, it will be said, "Well done, thou good and faithful servants." A. W. G.

### DR. KOCH AND HIS EXPERIMENT.

Since Dr. Koch read his paper on his experiments in inoculating against the ravages of the tubercle bacillus before the International Medical Convention convened in Berlin, the newspapers have been actively engaged in romancing, and the suffering public and ambitious M. D.'s are over-anxious to see the results; and we would not dampen their ardor, but would remind them that as yet they have nothing authoritative; and even further, Dr. Koch himself distinctly states "that he had not completed his experimentation, and could not give a positive or complete statement as to the ultimate result." Although suggestions and hints of possible discovery emanating from so scientific a source as Dr. Koch must inevitably have their weight, yet it behooves the profession at large to wait till the discoverer announces his work as complete before they should judge, for in questions so potent, the utmost conservatism is indicated, and rash experimentation is sure to be productive of evil, and will tend to injure what would by patience be beneficial. There is no question in our minds but that eventually some means will be discovered that will not only destroy the then existing ravages of bacillus tuberculosis, but also like conditions, and we think that more will be gained by patient and constant investigation than, to use a vulgar though pregnant expression, to "fly off the handle" at every suggestion of a new and wonderful discovery.

### TO OUR PATRONS.

THE RECORD, under its present management, has prospered wonderfully. At the time we took charge, last January, forty pages comprised the reading matter; now we have sixty pages. As an evidence of our prosperity with the advertising public, our books show an increase in that line equal to the reading matter. Our subscription has doubled, and is steadily increasing. We propose to continue the good work and make it the best medical journal in the South. After this issue THE RECORD will be printed on super-calendered book paper. We can assure our patrons that nothing will be left undone that money and brains can accomplish to make it the equal of any medical journal in this country. Our contributions will be from the most learned and successful men in the profession, and will cover all new and interesting subjects. No physician can afford to be without our journal if he wishes to keep up with his profession.

We want all the doctors to write us short reports of their interesting cases, for in this way we get the benefit of each other's experience. We desire to thank all of our subscribers and advertisers for their past kindness, and solicit a continuance of their patronage. Wishing you all the compliments of the season and continued success in your profession and business, I remain yours truly, DAN H. HOWELL,

Business Manager.

### AMERICAN PUBLIC HEALTH ASSOCIATION.

The Eighteenth Annual Meeting will be held in the city of Charleston, South Carolina, on December 16, 17, 18 and 19, at Hibernian hall. From the announcements that have been sent out, it will be the most important meeting that they have ever had.

THE CLINIC, Vol. I, No. 1, issued under the auspices of the Atlanta Medical College, edited by five of the students of the college, is a neat, well arranged and attractive little journal, and a very excellent medium for carrying out the purposes intended. The editors have plainly set forth, in the salutatory, what they have determined to do. We sincerely trust that they will "knock out the bung and let nature cut a caper." They have certainly made a very creditable commencement upon their journalistic career, and we bespeak for them and their journal the hearty endorsement and support of the profession generally. We wish them success, and extend them our hand, and bid them welcome to the fraternity of journalists.

### THE MEDICAL SOCIETY OF MACON, GA.

At a regular meeting of Macon Medical Society, the following officers were elected for the ensuing year: President, Dr. R. O. Cotter; Vice-President, Dr. H. J. Williams; Secretary and Treasurer, Dr. H. P. Derry; Corresponding Secretary, Dr. H. McHatton; Reporter, Dr. W. A. O'Daniel; Librarian, Dr. W. H. Holt.

### REMOVAL OF A PORTION OF LIVER FILLED WITH HYDATID CYSTS BY AN ELASTIC LIGATURE.

M. Terrillon communicates the case of a woman of fifty-three years, who suffered for several years with sharp pains in the right hypochondrium, coming on at certain marked periods. The diagnosis had been a cyst filled with calculi, and an explorative puncture, made in the swelling which existed in the right side, allowing one hundred grammes of hydatid liquid to escape, without diminishing the size of the swelling.

Laparotomy showed that he had to do with a diseased mass about the size of two fists, composed of a part of liver tissue, occupying the edge of the organ, and totally filled with little hydatid cysts.

A line of demarcation sharply separated the diseased tissue from the rest of the organ, which appeared quite healthy.

An elastic ligature was firmly applied at the point of this line. Almost immediately the ligature seemed to make a pedicle, and the part below the compressing force became turgescent. This was fixed outside the body by a few stitches of suture, and the rest of the wound closed. Iodoform antisepsis.

Little by little the part of the liver thus pediculated died, without producing any general reaction. After eleven days, as there was a slight elevation of temperature, M. Terrillon divided the pedicle. There remained a large wound which cicatrized in thirty days. In fact, the patient was completely cured and regained a normal weight and strength.

This is the first case on record where so large a resection of the liver was followed by cure.—*La Medicine Moderne; Times and Register; Pacific Record.*

